# Radical C─H‐Aroylation of Allenes via Cooperative Photoredox and *N*‐Heterocyclic Carbene Catalysis

**DOI:** 10.1002/anie.202511689

**Published:** 2025-07-30

**Authors:** Shyam Kumar Banjare, Lena Lezius, Armido Studer

**Affiliations:** ^1^ Chemistry Department, Organisch‐Chemisches Institut Universität Münster 48149 Münster Germany

**Keywords:** Acylation, Allenes, *N*‐heterocyclic carbene, Photocatalysis, Radical reactions

## Abstract

This study demonstrates the use of cooperative photoredox and *N*‐heterocyclic carbene (NHC) catalysis for sp^2^ C─H acylation of allenes. The cascade comprises oxidative generation of an allene radical cation from an allene, its nucleophilic trapping to the corresponding allyl radical and highly regioselective cross coupling by a concomitantly reductively generated NHC‐derived ketyl type radical. Ionic fragmentation of both the NHC and nucleophile ultimately yields the desired substituted allene. The organic photocatalyst, 4CzIPN, is highly effective in promoting both oxidative and reductive electron transfer steps. Tri‐ and tetra‐substituted allenes can be obtained in good yields through such a cascade. Mechanistic studies—including radical trapping, acylazolium reactions, and Stern–Volmer quenching—support the proposed mechanism. Moreover, follow‐up chemistry is conducted to demonstrate the synthetic value of the cascade products.

## Graphical abstract

Cooperative *N*‐heterocyclic carbene and photoredox catalysis for C─H benzoylation and esterification of allenes is presented. This reaction proceeds through the cross‐coupling of in situ generated ketyl and allyl radicals.

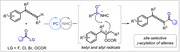

Allenes are highly valuable and versatile building blocks in organic synthesis.^[^ ^1^, ^2^, ^3^, ^4^, ^5^ ^]^ Their unique structure features a central sp‐hybridized carbon atom connected by two carbon─carbon double bonds (π‐bonds). Their reactivity is enhanced compared to other well‐known π‐systems, such as olefins and alkynes.^[^ ^6^, ^7^, ^8^, ^9^ ^]^ Notably, this structural motif is also present in various natural products and pharmaceutical compounds.^[^ ^10^, ^11^, ^12^, ^13^, ^14^ ^]^ Furthermore, allenes can be easily converted to multisubstituted furans.^[^ ^15^, ^16^ ^]^ However, the selective functionalization of sp^2^ C─H bonds in allenes remains an underdeveloped area.^[^ ^17^, ^18^ ^]^

Metal‐catalyzed activation of C─H bonds is a highly effective and versatile general strategy in organic synthesis for C─H functionalization.^[^ ^19^, ^20^ ^]^ Indeed, such an approach has also been applied to allenes, as shown by Ma who investigated the regioselective C─H arylation using palladium and rhodium catalysis through carbometalation/β‐hydride elimination pathways (Figure 1a).^[^ ^21^, ^22^ ^]^ Further advancements were achieved by Carreira who developed a method for direct γ‐selective allene C─H olefination, employing a directing group strategy.^[^ ^23^ ^]^ This approach operates through a concerted metalation‐deprotonation (CMD) pathway (Figure 1a). Radical‐based methods have also been explored.^[^ ^24^, ^25^, ^26^ ^]^ In this context, copper‐catalyzed oxidative amination of allene was developed using NFSI by Q. Zhang.^[^ ^27^ ^]^ Further, copper‐catalyzed hydrogen atom abstraction (HAA) processes with reactive heteroatom‐centered radicals have been recognized as reliable for generating allenyl radicals.^[^ ^28^ ^]^ This strategy was successfully employed for the γ‐selective C─H cyanation of allenes by Lin, Ma, and Liu (Figure 1b).^[^ ^29^ ^]^ As the cyanation step is mediated by the Cu‐catalyst, a few enantioselective cyanations were also disclosed. Applying a similar strategy, γ‐arylated allenes and alkynylated allenes can be obtained using aryl boronic acid and alkynes as the coupling partners.^[^ ^30^ ^]^ Further, Bao and Wu also realized metal‐catalyzed site‐selective cyanation^[^ ^31^, ^32^ ^]^ and alkynylation^[^ ^31^ ^]^ of allenes through radical intermediates.

**Figure 1:**
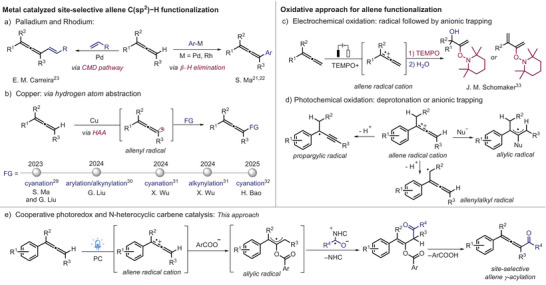
Different strategies for the functionalization of allenes.

As an alternative strategy for allene activation, Schomaker recently investigated electrochemical oxidation, successfully capturing the intermediately generated allene radical cation with the stable TEMPO radical (Figure 1c).^[^ ^33^ ^]^ Depending on the substituents, allenes possess relatively low oxidation potentials (e.g., 1.29 V vs. Fc/Fc^+^ for buta‐2,3‐dien‐2‐ylbenzene).^[^ ^34^, ^35^ ^]^ Unlike in the Schomaker case, the allene radical cations are generally trapped by nucleophiles to give allylic radicals (Figure 1d). This is the common reactivity mode as accumulation of allene radical cations and radicals in solution to achieve a selective radical‐radical cross coupling is not likely, unless one radical species is longer lived (persistent radical effect).^[^ ^24^ ^]^ A third reaction mode is the sp^2^ C─H deprotonation of the intermediate allene radical cation to generate the corresponding propargylic radical.^[^ ^36^ ^]^ Finally, if the allene radical cation carries an alkyl substituent, deprotonation at the α‐position of the alkyl group might occur to give an allenylalkyl radical. The relatively low oxidation potential of allenes allows them to be oxidized into their radical cations also through photochemical oxidation.^[^ ^37^, ^38^, ^39^, ^40^ ^]^ Over the past decades, photoredox chemistry has emerged as a powerful tool for organic transformations.^[^ ^41^, ^42^, ^43^ ^]^ However, the oxidation of allenes through photoredox catalysis is still not well developed.^[^ ^38^ ^]^ A key challenge is exerting control over the diverse reaction pathways available to intermediate allene radical cations (see Figure 1d).

Herein, we report cooperative photoredox/*N*‐heterocyclic carbene (NHC) catalysis^[^ ^44^, ^45^, ^46^ ^]^ for the formal direct C─H aroylation of 1,1‐di and trisubstituted allenes with aroyl fluorides (Figure 1e). These transformations proceed through regioselective cross‐coupling of ketyl radicals^[^ ^47^ ^]^ generated by single‐electron reduction of acylazoliums^[^ ^44^, ^45^, ^46^ ^]^ with allylic radicals that are derived from benzoate trapping of allene radical cations. The benzoate nucleophile is generated by the addition of the NHC to the in situ formed bisbenzoyl carbonate intermediate, which is derived from benzoyl fluoride, leading to the formation of an acyl azolium salt. Ionic NHC fragmentation followed by benzoate elimination eventually affords the C─H functionalized allenes. Notably, the selective γ‐acylation of allenes has not been accomplished to date, representing a significant advancement in synthetic chemistry. This process is of preparative value as acylated allenes are readily further transformed to multisubstituted furans.

We initiated our investigation using 1‐(buta‐2,3‐dien‐2‐yl)‐4‐methoxybenzene **1a** as the model substrate, which reveals an oxidation potential of 0.9 V versus Fc/Fc^+^ (refer to Scheme 3f for its cyclic voltammogram, CV). We employed benzoyl fluoride **2a** as the coupling partner, applying the organic photocatalyst 4CzIPN (*E_1/2red_ = +1.35 V vs. SCE)^[^ ^48^ ^]^ along with the triazolium salt **NHC‐1** (20 mol%) as the NHC precatalyst. The cascade reaction was best conducted in a mixture of dichloromethane and acetonitrile (1:1) at room temperature under blue LED irradiation (λ_max_ = 420 nm) for 12 h using Cs_2_CO_3_ as the base (2.0 equivalents) to afford the desired product **3** in 69% yield (Table 1, entry 1). Of note, the allene product derived through benzoylation of the methyl substituent was not observed.

**Table 1:** Variation of reaction conditions.^a)^

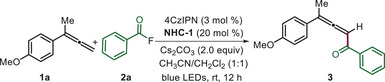		
Entry	Deviation from the standard reaction conditions	^b)^Yield of **3** (%)
1	none	69
2	no photocatalyst	<5
3	no N‐heterocyclic carbene (NHC)	<5
4	[Ir[dF(CF_3_)ppy]_2_(dtbbpy)]PF_6_ catalyst instead of 4CzIPN	40
5	[Ir(ppy)_3_] catalyst instead of 4CzIPN	<5
6	Ru(bpy)_3_ (BF_4_)_2_ catalyst instead of 4CzIPN	<5
7	[Acr‐Mes]CIO_4_ catalyst instead of 4CzIPN	<5
8^c)^	other solvents instead of CH_3_CN	<5
9	without CH_2_Cl_2_ only CH_3_CN	49
10	without CH_3_CN only CH_2_Cl_2_	53
11^d)^	other bases instead of Cs_2_CO_3_	<5
12	other **NHCs‐2–4** instead of **NHC‐1**	<5
13	**NHC‐5** instead of **NHC‐1**	64
14	with **2b** instead of **2a**	66
15	with **2c** instead of **2a**	43
16	with **2d** instead of **2a**	51
17	with **2e** instead of **2a**	<5
18	with **2f** instead of **2a**	<5
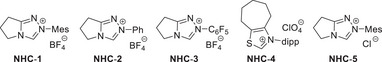		
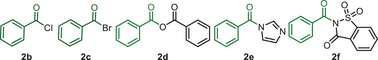		

^a)^ Reaction conditions: **1a** (0.1 mmol), **2a** (0.25 mmol), PC (3 mol%), NHC (20 mol%), base (2.0 equivalents). CH_3_CN/CH_2_Cl_2_ (2 mL) under irradiation with blue LEDs.

^b)^ Isolated yield.

^c)^ *N*,*N*‐dimethylformamide, dimethyl sulfoxide, toluene, hexane.

^d)^ K_2_CO_3_, Na_2_CO_3_, CaCO_3_, K_2_HPO_4_, Et_3_N, DBU.

Additionally, we conducted control experiments, including reactions without a photocatalyst and the triazolium salt **NHC‐1**. In these cases, only trace amounts of the product were observed, showing the necessity of both the NHC and also the photoredox catalyst (Table 1, entries 2,3).

Replacing 4CzIPN by [Ir(dF(CF_3_)ppy)_2_(dtbbpy)]PF_6_ provided a significantly reduced yield (40%, Table 1, entry 4). However, other photocatalysts, such as Ir(ppy)_3_ and Ru(bpy)_3_, as well as an acridinium‐based catalyst, did not work (Table 1, entries 5–7). We also screened various solvents^[^ ^49^ ^]^ and noted for aprotic polar solvents such as DMF and DMSO, as well as for the less polar solvents benzene and hexane that C─H benzoylation of **1a** did not proceed (Table 1, entry 8). However, in pure dichloromethane or pure acetonitrile a 49% and 53% yield of **3** was obtained (Table 1, entries 9,10). Additionally, different metal salts and organic bases, such as triethylamine and 1,8‐diazabicyclo[5.4.0]undec‐7‐ene, were screened. However, all these alternatives proved ineffective (Table 1, entry 11). Various NHC precursors, including triazolium and thiazolium salts, were also tested in place of **NHC‐1**. Most of them were inefficient in providing the targeted product **3** (Table 1, entry 12). As expected, the triazolium chloride salt **NHC**‐**5** carrying a different counter anion as compared to **NHC**‐**1** delivered **3** in a good yield (64%, Table 1, entry 13). Next, we explored a range of NHC acylation reagents as replacements for benzoyl fluoride. Among these, benzoyl chloride (**2b**), benzoyl bromide (**2c**), and benzoic acid anhydride (**2d**) afforded the desired product **3** in moderate to good yields (Table 1, entries 14–16). However, the *N*‐benzoyl derivatives **2e** and **2f** turned out to be inefficient (Table 1, entries 17,18). We also observed a side product derived from targeted allene **3**, which, upon further oxidation and trapping, gives rise to the 1,1‐diaroylated allene **32** in trace amounts (see Scheme 2b). Additionally, enol ester **VII** has been detected by HRMS as an additional side product in this and most of the following transformations (see Scheme 3).

Following optimization of the reaction conditions, the scope of the method was investigated (Scheme 1). The larger‐scale synthesis of **3** was possible without compromising yield to a large extent. The meta‐methoxyphenyl allene **1b** reacted with lower efficiency (see **4**), but the good yield was restored for the more readily oxidizable allene **3c** to give **5** (65%). The protocol is also applicable to 1,1‐diaryl‐substituted allenes as demonstrated by the successful syntheses of **6** and **7**.

**Scheme 1:**
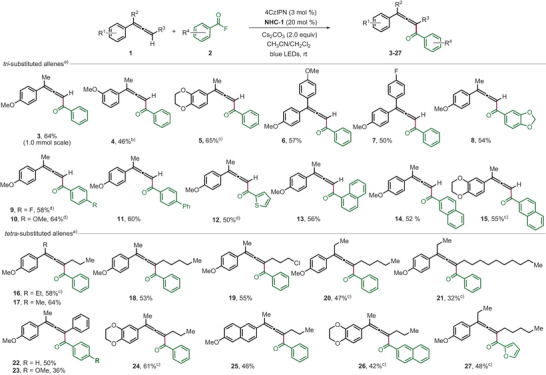
Scope of di‐ and trisubstituted allenes also varying the aroyl fluoride component. ^a)^The reaction was performed with **1** (0.10 mmol) and **2** (0.25 mmol) under an argon atmosphere for 12 h and isolated yields are provided. ^b)^Reaction time was 24 h. ^c)^Reaction time was 6 h. ^d)^Conducted with the corresponding aroyl chloride.

We then varied the acylation reagent keeping allene **1a** as reaction partner. Benzo[d][1,3]dioxole‐5‐carbonyl fluoride **2** **g** provided **8** in 54% yield. With para‐methoxy and para‐fluoro‐substituted benzoyl chloride as benzoyl precursors, the desired allenes **9** and **10** were obtained in 58% and 64% yields, respectively. Biphenyl‐4‐carbonyl fluoride **2j** proved effective, providing **11** in 60% isolated yield. Thiophene‐2‐carbonyl chloride **2k** was found to be an eligible acyl donor resulting in a 50% yield of allene **12**. Furthermore, we expanded the reaction scope to include various naphthyl derivatives, delivering the corresponding products in 52%–56% yields (**13**, **14,** and **15**).

Next, we tested whether tetra‐substituted allenes can be accessed through this novel approach. Pleasingly, under the optimized reaction conditions, we were able to obtain the tetra‐substituted products **16** and **17** in 58% and 64% yields. Selectivity was excellent as the product derived from a C─H benzoylation at the methyl side chain was not observed. The impact of the aliphatic chain was evaluated by replacing the n‐propyl by longer linear alkyl chains. Products were formed in all cases and we noted a decrease in the yield as a function of the alkyl chain length (see **18**, **19**, **20,** and **21**). The diarylalkyl substituted allene **1l** reacted with **1a** and **1i** to provide the tetra‐substituted allene **22**, **23** in moderate yield. Reaction was general for readily oxidizable trisubstituted allenes carrying electron‐rich dimethoxyaryl and naphthyl substituents, yielding products **24** and **25** in 61% and 46%, respectively. Moreover, both 2‐naphthoyl fluoride **2m** and furan‐2‐carbonyl fluoride **2n** were tolerated, affording the targeted allenes **26** and **27** in moderate yields with high selectivity with respect to the sp^2^ C─H aroylation.

Studies were continued by addressing the allene sp^2^ C─H alkoxycarbonylation using dimethyl dicarbonate (**28**) as C1‐carbon source (Scheme 2a). Under the conditions optimized for the aroylation, methoxy carbonylation of allene **1f** to give **29** was achieved. However, the regioisomeric propargyl compound **29′** that could not be separated was formed in equal amounts (55% combined yield). A similar reaction outcome was noted for the methoxy carbonylation of allene **1j** to give **30** along with its regioisomer **30′**. For allene **1o** the propargylic regioisomer **31′** was obtained as the major product (1:2) in 47% combined yield. We assume that due to higher basicity of the methoxide that is formed as a byproduct in the reaction of the NHC with dimethyl dicarbonate, deprotonation of the intermediate allene radical cation to give the propargyl radical (see Figure 1d) becomes competitive with the trapping by the nucleophile.

**Scheme 2:**
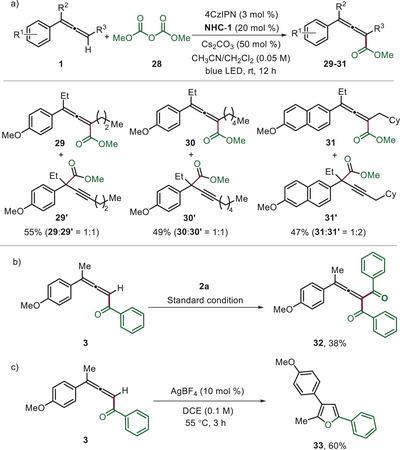
a) Scope for alkoxycarbonylation of trisubstituted allene. The reaction was performed with **1** (0.10 mmol) and **28** (0.25 mmol) under an argon atmosphere, and yields were isolated after 12 h. b) Reaction was performed with **3** (0.10 mmol) and **2a** (0.25 mmol) under an argon atmosphere for 12 h. c) Synthesis of furan from benzoyl allene **3** (0.10 mmol) and AgBF_4_ (10 mol%), dichloroethane 1.0 mL at 55 °C.

Notably, 1,1‐diaroylated allene **32** could be prepared in moderate 38% yield after subjecting the mono‐benzoylated allene **3** to the reaction conditions (Scheme 2b). This further indicates that overaroylation can reduce the yield of the C─H‐functionalization of 1,1‐disubstituted allenes. To document the synthetic value of the aroylated product allenes, **3** was successfully converted to the trisubstituted furan **33** using a catalytic amount of silver tetrafluoroborate (Scheme 2c). This interesting cascade reaction comprises a 1,2‐aryl migration prior to the cyclization step and the furan was formed with exclusive regioselectivity.^[^ ^50^ ^]^ The limitation of this C─H aroylation method lies in its restriction to the functionalization of electron‐rich allenes that can easily be oxidized to their radical cations. Thus, 1‐aryl‐substituted 1‐methyl‐allenes, such as those containing a 1‐para‐chlorophenyl (**1p**), a para‐cyanophenyl‐ (**1q**) or even the phenyl‐substituted congener (**1s**) were not eligible substrates and the starting allenes remained unreacted. Furthermore, sterically bulky *tert*‐butyl trisubstituted allenes (**1r**) did not react and also mono‐substituted para‐methoxyphenyl allene **1t** failed to yield the desired product (see SI for all failed substrates, Note 4).

Finally, mechanistic studies were conducted. The reaction was performed using an isolated acylazolium ion intermediate under optimized conditions, resulting in trace product formation. However, upon addition of 1.0 equivalent of benzoic acid reactivity could be restored and **3** was formed in 31% yield, showing that an acylazolium ion is a likely intermediate of the transformation (Scheme 3a). Furthermore, this experiment also revealed that benzoic acid is required in this cascade to trap the cation. To evaluate the efficiency of cation trapping, various deprotonated acids were tested in the reaction with **1a** using the isolated acylazolium ion intermediate as the reaction partner. While the benzoate provided the targeted product allene, deprotonated phosphoric acid, acetic acid, and tosic acid did not work (traces). The reaction was completely suppressed in the presence of 2.0 equivalents of TEMPO (2,2,6,6‐tetramethylpiperidine‐*N*‐oxyl), and the formation of an acyl‐TEMPO adduct was confirmed by high‐resolution mass spectrometry (HRMS). This observation suggests that radicals are likely involved in the cascade process (Scheme 3b).^[^ ^51^ ^]^ The reaction of **1a** with **2a** was repeated under the standard conditions in the presence of the radical acceptor methyl 2‐((phenylsulfonyl)methyl)acrylate. Formation of product **3** was significantly suppressed, and the allenylation product was detected by mass spectrometry, suggesting the involvement of radical character at the allene C3‐position during the transformation (Scheme 3c). Conducting the reaction in perdeuterated acetonitrile resulted in no deuterium incorporation, indicating that solvent‐derived hydrogen atoms or protons do not participate in the reaction mechanism (Scheme 3d). To evaluate the relative reactivity of di‐ and trisubstituted allenes, a competition experiment between **1a** and **1** **g** was performed. The results revealed that the 1,1‐disubstituted allene reacts more efficiently, with a product ratio of 3:1 (Scheme 3e). Of note, the Stern‐Volmer quenching studies showed preferable oxidation of **1** **g** over **1a** (see Scheme 3f). Thus, the initial allene oxidation does likely not correspond to the rate‐determining step of this sequence. Cyclic voltammetry studies suggest that the photocatalyst can oxidize tri‐substituted allenes more easily than di‐substituted allenes. Furthermore, Stern–Volmer fluorescence quenching experiments indicate that the photocatalytic cycle proceeds via a reductive quenching pathway, as the allenes quench the excited‐state photocatalyst more efficiently than the acylazolium salt (Scheme 3f).

**Scheme 3:**
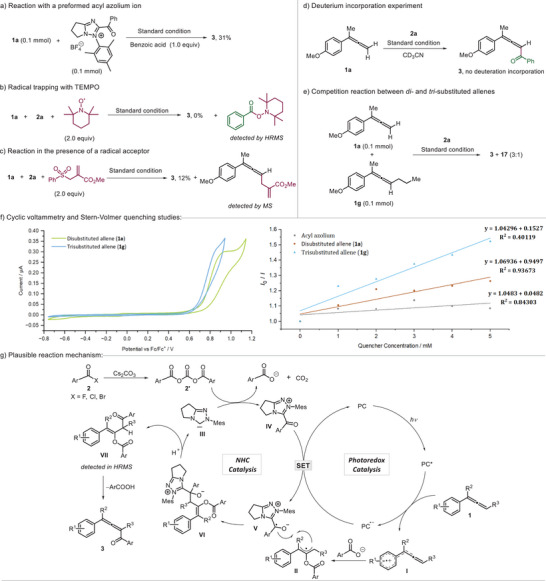
Mechanistic studies and proposed mechanism.

Based on these studies and literature reports,^[^ ^46^, ^52^, ^53^, ^54^ ^]^ a plausible reaction mechanism is depicted in Scheme 3g. Under blue light irradiation, allene **1** gets oxidized to its radical cation **I** by the excited 4CzIPN photocatalyst [E_1/2_ (4CzIPN*/4CzIPN^•−^) = 1.35 V vs. SCE].^[^ ^48^ ^]^ This allene radical cation is then trapped by the carboxylic acid that is formed during acyl azolium salt formation from **2**, Cs_2_CO_3_ and the NHC **III** via **2′** to afford the transient allyl radical **II**. The acylazolium intermediate **IV** (E_1/2_ = –0.81 V vs. SCE for Ar = Ph)^[^ ^55^ ^]^ can then be reduced by the radical anion of the photocatalyst (E_1/2_ (4CzIPN/4CzIPN^•−^) = –1.21 V vs. SCE)^[^ ^48^ ^]^ to give the ketyl type radical **V**. Selective cross coupling of the ketyl **V** with the allyl radical **II** steered by the persistent radical effect^[^ ^26^ ^]^ leads to intermediate **VI**. The regioselectivity is likely governed by steric effects. NHC fragmentation completes the NHC catalysis cycle to give the enol ester **VII** that could be detected by HRMS. Elimination of the aromatic carboxylic acid probably through an E1cB‐type elimination process finally provides the isolated C─H aroylated allene **3**.^[^ ^56^ ^]^

In summary, we have developed a protocol for the selective acylation of allenes under photoredox conditions. The cascade process operates through cooperative NHC and photoredox catalysis. Di‐ and tri‐substituted allenes can be C─H aroylated in moderate to good yields. Reactions proceed through allene radical cations that can express diverse reactivity. Despite several options, selectivity towards formation of the di‐ as well as a tetra‐functionalized sp^2^ C─H allene functionalization product was excellent in most cases. Aroyl fluorides and also aroyl chlorides serve as the acyl donors in these transformations. With dialkyl dicarbonates in place of the acid halides, allene C─H alkoxycarbonylation can be achieved. However, in this latter case, selectivity was not complete and α‐alkynylated esters were formed as side products. Comprehensive mechanistic studies were conducted to support the proposed reaction pathway. The introduction of these reactions broadens the scope of photoredox and NHC cooperative catalysis, as well as allene functionalization. Incorporating these findings will advance both the understanding and application of radical NHC catalysis as researchers continue to explore new methodologies.

## Author Contributions

S.K.B. ran the experiments. L.L. performed the fluorescence and cyclic voltammetry measurements. S.K.B. and A.S. designed the experiments. All authors approved the final version of the manuscript.

## Conflict of Interests

The authors declare no conflict of interest.

## Data Availability

The data that support the findings of this study are available in the Supporting Information of this article.
